# A review of MASLD-related hepatocellular carcinoma: progress in pathogenesis, early detection, and therapeutic interventions

**DOI:** 10.3389/fmed.2024.1410668

**Published:** 2024-06-04

**Authors:** Yang Ma, Jinguo Wang, Wenping Xiao, Xiaoming Fan

**Affiliations:** ^1^Department of Human Anatomy, School of Basic Medicine, Guilin Medical University, Guilin, China; ^2^School of Public Health, Guilin Medical University, Guilin, China

**Keywords:** metabolic dysfunction-associated steatotic liver disease, hepatocellular carcinoma, inflammatory response, oxidative stress, the intestinal microbiome

## Abstract

The incidence of metabolic dysfunction-associated steatotic liver disease (MASLD) is continuously rising, evolving into a global health challenge. Concurrently, cases of hepatocellular carcinoma (HCC) associated with MASLD are also on the increase. Although traditional risk factors such as age, gender, and metabolic factors play significant roles in the development of HCC, it cannot be overlooked that MASLD, triggered by changes in modern lifestyle and dietary habits, may also exacerbate the risk of HCC, and this phenomenon is common even among non-obese individuals. Regrettably, MASLD often fails to receive timely diagnosis, resulting in a limited number of patients receiving HCC surveillance. Moreover, there is currently a lack of clear definition for the target population for surveillance beyond patients with cirrhosis. Consequently, MASLD-related HCC is often detected at a late stage, precluding the optimal timing for curative treatment. However, our understanding of the pathogenesis and progression of HCC remains limited. Therefore, this paper reviews relevant literature from recent years, delving into multiple dimensions such as pathogenesis, surveillance and diagnosis, prevention, and treatment, aiming to provide new ideas and directions for the prevention and treatment of MASLD-related HCC.

## 1 Introduction

Primary hepatic carcinoma ranks as the sixth most common cancer and the third leading cause of cancer deaths globally. Hepatocellular carcinoma (HCC) accounts for 75%−85% of these cases. In 2020, ~900,000 new cases of primary liver cancer were reported worldwide, leading to 830,000 deaths, indicating a close proximity between the incidence and mortality rates (1). Multiple risk factors contribute to HCC, including viral hepatitis, aflatoxin exposure, exposure to certain chemicals, and metabolic dysfunction-associated steatotic liver disease (MASLD) (2). In the past, viral hepatitis was the primary risk factor for HCC. However, with improvements in lifestyle and living conditions, the prevalence of MASLD has been increasing, posing a significant global public health challenge (3). Concurrently, the incidence of MASLD-related HCC is also on the rise. Although the incidence of MASLD-related HCC is lower compared to other causes such as hepatitis B-related HCC, the prevalence of MASLD surpasses that of other liver diseases. Furthermore, MASLD-related HCC is often diagnosed at an advanced stage, making curative treatment unfeasible (4). Therefore, there is an urgent need to raise global awareness and conduct thorough research on the underlying mechanisms to mitigate the impending burden of MASLD-related HCC.

## 2 Epidemiological investigation and association analysis of MASLD and HCC

The worldwide occurrence and frequency of MASLD are rapidly on the rise, with a global estimation of ~25% for its prevalence (5). Notably, the majority of metabolic dysfunction-associated steatohepatitis (MASH) cases are expected to increase by 56% between 2016 and 2030 in China, France, Germany, Italy, Japan, Spain, the United Kingdom, and the U.S. (6). MASLD is characterized as a clinicopathological syndrome marked by hepatic steatosis, associated with factors such as insulin resistance (IR) (7), autophagy (8), and gut microbiota (9), and obesity (10). These factors collectively contribute to chronic inflammation, altered lipid metabolism, and ultimately, the promotion of HCC development. Given that primary liver cancer ranks as the sixth most common cancer and the third leading cause of cancer death worldwide (11), these findings highlight the substantial risk of liver cancer. Studies suggest that the incidence of MASLD-related HCC may increase alongside modern living standards and lifestyle changes. For instance, 35% of the U.S. population was obese in 2012, with projections estimating that 48.9% will be obese by 2030 (12). This obesity epidemic is likely to parallel an increase in the incidence of MASLD-related HCC. A similar crisis is unfolding in China, where the prevalence of MASLD is expected to rise in tandem with the obesity and diabetes epidemics. Although most patients with MASLD present with simple steatosis without advanced liver disease, a subset of these patients may progress to MASH, leading to cirrhosis and HCC. From 2016 to 2030, an 82% increase in HCC incidence is projected for China (6). Additionally, a significant proportion of MASLD patients without cirrhosis may develop HCC (13), underscoring the importance of further examining the link between cirrhotic MASLD, non-cirrhotic MASLD, and HCC in our discussion.

### 2.1 The association between MASLD and HCC in cirrhosis

It is now widely recognized that cirrhosis serves as a critical risk factor for the emergence of HCC in MASLD patients, with an annual incidence rate of 10.6 per 1,000 person-years among those with cirrhosis caused by MASH (14). The primary cause of MASLD is overnutrition, resulting in the enlargement of fat deposits and ectopic fat accumulation (15). In this scenario, the infiltration of macrophages into visceral adipose tissue compartments gives rise to a proinflammatory state, promotes insulin resistance, and imbalances in lipid metabolism, leading to the formation of lipotoxic lipids. These lipids contribute to cellular stress, including oxidative stress and endoplasmic reticulum stress, and trigger inflammasome activation, apoptotic cell death, inflammatory stimulation, tissue regeneration, and fibrosis, ultimately leading to cirrhosis (16). Notably, the large vesicular steatosis observed in hepatocytes disappears in advanced cirrhosis, and the underlying mechanism for this fat disappearance remains under investigation (17). A prevailing hypothesis suggests that the physiological characteristics of cirrhosis may either directly or indirectly affect the ability of hepatocytes to uptake triglycerides, resulting in abnormalities in the flow of fat-forming molecules to the subendothelial hepatocytes, thus impacting hepatocyte permeability (18). Nevertheless, the precise mechanisms linking MASLD and HCC in the context of cirrhosis remain elusive and require further intensive investigation.

### 2.2 The association between non-cirrhotic MASLD and HCC

In contrast to hepatitis virus-driven HCC, a significant proportion of patients with MASH develop HCC in the absence of cirrhosis (19, 20). The exact mechanisms underlying this phenomenon remain partly unclear but are thought to be related to pathophysiological changes associated with lipotoxicity, bile acid (B.A.) signaling, and inflammation (21). It has been shown that serum B.A. concentrations are elevated in patients with advanced MASH, and B.A. accumulation can induce parenchymal injury (22). This, in turn, promotes the progression of MASLD to HCC. Among patients without cirrhosis who progress to HCC, the highest risk is observed in males over 65 years old with a history of smoking, type 2 diabetes, and elevated levels of alanine aminotransferase (ALT) (23). Furthermore, heightened levels of ALT and indicators of liver inflammation have been independently correlated with an increased risk of HCC [hazard ratio (HR) 6.80, 95% CI: 3.00–15.42; *p* < 0.001] among individuals with non-cirrhotic MASLD. This increased risk is attributed to the influence of a proliferative environment and inflammation on tumor development (24). This highlights the critical role of metabolic and inflammatory pathways in the pathogenesis of HCC in patients without cirrhosis. However, the exploration of other potential mechanisms remains an essential area for further research.

## 3 The mechanisms underlying the advancement of MASLD to HCC

**Figure 1:**
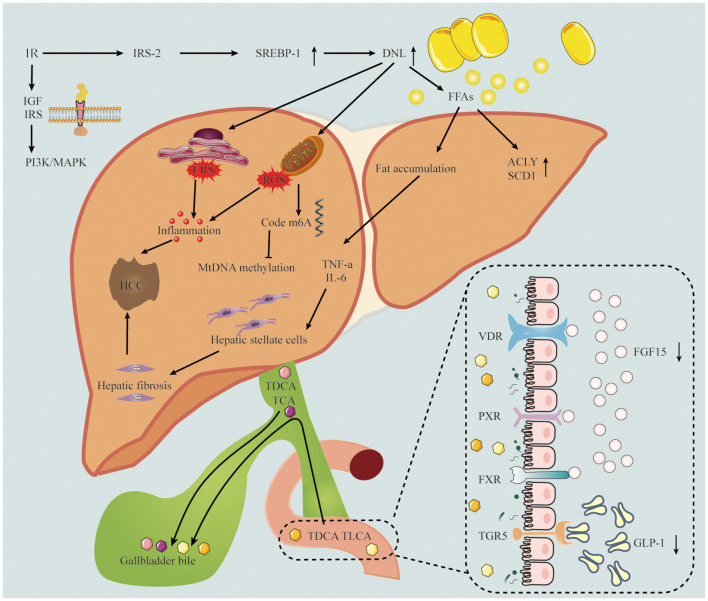
The pathogenesis of MASLD-related HCC includes insulin resistance, inflammation, oxidative stress and gut microbiota. The down regulation of IRS-2 leads to the over expression of SREBP-1 and the up-regulation of DNL, which leads to inflammatory response and oxidative stress. TDCA and TLCA in intestinal epithelial cells interact with bile acid receptors, resulting in FGF-15 and GLP-1 decline. IRS-2, insulin receptor substrate 2; SREBR-1, sterol regulatory element-binding protein 1; DNL, *de novo* lipogenesis; FFAs, free fatty acids; IGF, insulin-like growth factor; ACLY, ATP citrate lyase; SCD1, stearoyl coenzyme A desaturase-1; TNF-a, tumor necrosis factor alpha; IL-6, Interleukin 6; TDCA, taurodeoxycholic acid; TLCA, taurolithocholic acid; TCA, taurocholic acid; VDR, vitamin D receptor; PXR, pregnane X receptor; FXR, Farnesoid X nuclear receptor; TGR5, transmembrane G protein-coupled receptor 5; FGF15, fibroblast growth factor 15; GLP-1, glucagon-like peptide-1.

The progression of MASLD to HCC is facilitated by a range of mechanisms, as illustrated in Figure 1.

### 3.1 Insulin resistance

The initiation of MASLD/MASH is marked by the accumulation of fat in the liver, with IR being the pivotal pathogenic event leading to hepatic steatosis. Insulin typically promotes anabolic processes in visceral adipose tissue; however, adipocyte *de novo* lipogenesis is diminished during insulin resistance (25). Furthermore, the expression of carbohydrate response element binding protein beta (ChREBP beta), which positively regulates *de novo* lipogenesis, is notably low in the visceral adipose tissue of individuals with obesity or those with both obesity and type 2 diabetes. Moreover, the level of ChREBP beta in visceral adipose tissue is inversely associated with the severity of hepatic steatosis in these individuals (26). In the context of IR, the downregulation of insulin receptor substrate 2 (IRS-2) contributes to the overexpression of sterol regulatory element-binding protein 1 (SREBP-1) (27). This condition leads to an upregulation of *de novo* lipogenesis (DNL), crucial for promoting lipid storage in the liver. Moreover, β-oxidation of FFAs is inhibited in states of IR, further exacerbating hepatic lipid accumulation (28). Insulin and insulin-like growth factor-1 (IGF-1) are growth factors known to inhibit cell apoptosis. The synthesis and bioactivity of IGF-1 increase in the presence of IR, and elevated levels of IGF-1 stimulate cell proliferation while inhibiting apoptosis. This effect significantly elevates the risk of hepatocellular carcinogenesis (29). Insulin receptor substrate (IRS) molecules, upon binding to insulin or IGF, activate the phosphatidylinositol 3-kinase (PI3K)/protein kinase B (AKT) pathway and the mitogen-activated protein kinase (MAPK) pathway, promoting hepatocarcinogenesis (30). This intricate interplay of metabolic pathways underscores the complex relationship between insulin resistance and the progression of MASLD/MASH to HCC.

### 3.2 Inflammatory response

The inflammatory response is a critical hallmark of MASLD, with inflammation being driven by multiple intra- and extrahepatic factors (31, 32). A high-calorie diet, obesity, certain lifestyles, and genetic predispositions all contribute to the risk of developing MASLD. Hepatocyte overload and an increase in *de novo* lipogenesis lead to excessive lipid accumulation within hepatocytes. This lipid overload induces lipotoxicity, resulting in endoplasmic reticulum (E.R.) stress, oxidative stress, reactive oxygen species (ROS) production, and mitochondrial damage. In response to stress, hepatocytes release pro-inflammatory mediators and damage-associated molecular patterns (DAMPs), leading to the activation and infiltration of immune cells, which further exacerbates hepatocyte damage. Various forms of cell death, along with hepatocyte senescence, trigger a more pronounced immune response (33). Bile duct cells may also contribute to the inflammatory milieu by releasing inflammatory mediators. Moreover, hepatic inflammation extends to several extrahepatic systems, including adipose tissue, the intestine, skeletal muscle, and bone marrow (34). Necroinflammation, hepatocyte death, and oxidative stress significantly increase the risk of hepatocarcinogenesis. For instance, oxidative stress in hepatocytes activates inflammatory pathways, such as those mediated by STAT1 and STAT3, which are known pro-inflammatory transcription factors. Elevated levels of STAT1 are implicated in inducing MASH-related inflammation, while high levels of STAT3 independently drive the development of HCC in mice. This illustrates the intricate connection between inflammation and carcinogenesis in the progression from MASH to HCC (35). Such findings highlight the complex interplay between metabolic dysregulation and immune responses in the pathogenesis of MASLD and its progression to HCC.

### 3.3 Oxidative stress

Oxidative stress (O.S.) is defined as a condition where the production of reactive oxygen species (ROS) by the body surpasses the detoxification ability of antioxidants. In simpler terms, it represents a pathological state characterized by an imbalance in the body's antioxidant system, leading to increased oxidation (36). Oxidative stress stands as a pivotal hallmark of MASLD. The free radicals responsible for inducing O.S. play a crucial role in the advancement of MASLD, primarily through disrupting the process of lipid peroxidation via fatty acid mitochondrial oxidation, alongside instigating the release of cytokines, which subsequently ignite inflammation (37). Consequently, O.S. inflicts damage upon hepatocytes. The lipid peroxidation process initiates when free radical ROS abstract hydrogen atoms from unsaturated fatty acids, sparking a deleterious chain reaction. This chain reaction not only leads to the rupture of cell membranes but also results in the generation of reactive metabolites capable of causing cellular dysfunction. Furthermore, lipid peroxidation and its ensuing products stimulate hepatic stellate cells and promote the upregulation of pro-inflammatory cytokines (38). Hepatic iron overload is commonly observed in patients with MASLD. Due to the Fenton reaction catalyzed by iron, highly reactive hydroxyl radicals are generated from hydrogen peroxide (39, 40). Excessive hepatic iron can exacerbate oxidative stress and inflammation, and elevated iron levels can lead to increased ROS production. The occurrence of ferroptosis largely depends on the interconnected signals among subcellular organelles such as mitochondria, lysosomes, and peroxisomes. These organelles coordinate the regulation of ROS production and lipid oxidation during ferroptosis (41). Additionally, these processes have the potential to trigger cellular pathways leading to necrosis and apoptosis (42), which could culminate in liver fibrosis and, ultimately, HCC. This intricate cascade underscores the significant role oxidative stress plays in the progression from MASLD to more severe hepatic conditions.

### 3.4 Gut microbiota

The transport of blood from the gut to the liver, despite an efficient multi-layered intestinal barrier, is accompanied by metabolites and products of the intestinal microbiota, a process often referred to as microbiota-associated molecular patterns (43). In the liver, Kupffer cells serve as an effective “bacterial firewall,” averting bacterial infection while maintaining low levels of inflammation under physiological conditions (44). Additionally, the expression of specific bile acid synthases is influenced by the gut microbiota. Among MASLD's crucial bile acid receptors are the Farnesoid X nuclear receptor (FXR) and the transmembrane G protein-coupled receptor 5 (TGR5) (45). FXR has the capability to bind to ligands and form dimers with the retinoid X receptor, thereby regulating the transcription of target genes (46). These target genes encompass a variety of functions, including bile acid metabolism/transport, lipid metabolism, and glucose metabolism (47). Emerging studies have shown that gut flora may precipitate MASLD by modulating bile acid metabolism, thereby playing a role in the progression from MASLD to HCC (48–50). Increased intestinal permeability is a characteristic of cirrhosis, which subjects the liver to a substantial load of bacteria and bacterial components. Furthermore, within the gut microbiota-bile acid axis, secondary bile acids can enhance Toll-like receptor 2 (TLR2) expression on hepatic stellate cells and elevate levels of the TLR2 agonist lysophosphatidic acid. This, in turn, promotes the secretion of senescence-associated secretory phenotype (SASP) factors, thereby accelerating tumor progression (51). Additionally, the metabolism of bile acids by gut microbiota can influence liver tumor growth by modulating the hepatic expression of CXCL16, which is instrumental in recruiting natural killer T (NKT) cells (52). This intricate interplay between gut microbiota and liver pathology underscores the complex mechanisms by which gut-liver axis dysregulation contributes to the pathogenesis and progression of liver diseases, including MASLD and HCC.

### 3.5 Genetics and epigenetics

Polygenic risk scores based on genetic background are becoming fundamental for monitoring MASLD and assessing clinical risk, particularly for HCC. The *PNPLA3, TM6SF2*, and *MBOAT7* genes play crucial roles in the development and progression of MASLD. These genes affect lipid droplet accumulation, mitochondrial functionality, and metabolic reprogramming, leading to HCC (53). Epigenetics involves alterations in gene expression levels due to changes that do not affect the DNA sequence itself, primarily through the regulation of gene transcription or translation processes, thereby influencing their function and properties. The metabolic ecology of tumors is intricate, involving reprogramming of metabolism to re-establish the tumor microenvironment conducive for survival and proliferation. This reprogramming encompasses DNA methylation, histone acetylation, and N6-methyladenosine (m6A) modifications. Among these, epigenetic alterations, such as mitochondrial DNA (mtDNA) methylation, may occur during the development of MASLD. Mitochondria serve as both a major source and target of reactive oxygen species (ROS), with the mitochondrially encoded NADH dehydrogenase six genes being a site of mitochondrial methylation in MASLD (54). m6A is recognized as the most prevalent internal RNA modification in eukaryotes, playing a pivotal role in lipid metabolism, hepatocyte inflammation, and the progression of MASLD to liver tumorigenesis and metastasis. Excessive m6A modification can lead to increased expression of citrate lyase (ACLY) and stearoyl-CoA desaturase (SCD1). Furthermore, *in vitro* targeting of METTL3/14 has been shown to elevate the protein levels of ACLY and SCD1, resulting in increased production of triglycerides and cholesterol and accumulation of lipid droplets (55). A comparative study identified that 44 miRNAs were differentially expressed in the liver of MASLD patients vs. a healthy liver. Notably, research in MASH consistently observed a downregulation of liver-specific miR-122 and an upregulation of miR-34a (56). These changes in miRNA-targeted genes play crucial roles in hepatic energy metabolism, inflammation, cell regeneration, and fibrotic signaling, driving the progression from MASLD to hepatic fibrosis and subsequently to HCC (57). This highlights the significant impact of epigenetic mechanisms in the pathogenesis and progression of liver diseases, underlining the importance of understanding these processes for developing targeted therapies. The pathogenesis of MASLD-related HCC were showed in Table 1.

**Table 1:** The pathogenesis of MASLD-related HCC.

**Machine**	**Related molecules or genes**	**Role in pathogenesis**	**Implication for MASLD-HCC**	**Source magazine**	**References**
IR (insulin resistance)	IRS-2, SREBP-1	Promotes lipogenesis	Contributes to hepatocarcinogenesis	Diabetes Care	(28)
	IGF-1	Stimulates hepatocyte proliferation	Increases risk of HCC	Clinical Endocrinology and Metabolism	(29)
	IRS, IGF	Activates PI3K and MAPK signaling pathways	Promotes development of HCC	Journal of Hepatology	(30)
OS (oxidative stress)	Free radical	Causes liver cell injury and lipid peroxidation	Leads to HCC	Nature Immunology	(35)
	Mitochondrial oxidation				
Inflammatory response	STAT1, STAT3	Induction of inflammation in MASH	Augments progression to HCC	Clinica Chimica Acta	(38)
Intestinal flora	FXR, TGR5	Regulates bile acid metabolism and glucose metabolism	Alters bile acid signaling contributing to hepatocarcinogenesis	European Journal of Pharmacology	(45)
	TLR2	Promotes SASP secretion	Facilitates inflammation and tumor progression	Cancer Discovery	(51)
	CXCL16	Increases NKT cell recruitment	Regulates liver tumor growth	Science	(52)
Epigenetics	m6A, ACLY, SCD, METTL3/14	Alters lipid and glucose metabolism	Leads to hepatic fibrosis and potentially HCC	Molecular Therapy	(55)
Genetics	PNPLA3, TM6SF2, MBOAT7	Lipid droplet accumulation, mitochondrial functionality, and metabolic reprogramming	Leads to HCC	Cellular and Molecular Gastroenterology and Hepatology	(53)

IR, insulin resistance; OS, oxidative stress; IGF-1, insulin-like growth factor 1; PI3K, phosphoinositide 3-kinase; MAPK, mitogen-activated protein kinase; STAT, signal transducer and activator of transcription; MASH, metabolic dysfunction-associated steatohepatitis; FXR, Farnesoid X receptor; TGR5, G protein-coupled bile acid receptor; SASP, senescence-associated secretory phenotype; NKT, natural killer T; m6A, N6-methyladenosine; ACLY, ATP citrate lyase; SCD1, stearoyl-CoA desaturase-1.

## 4 Monitoring the evolution and diagnosing MASLD-HCC

MASLD represents a potentially severe liver disease, leading to significant healthcare costs, economic losses, and diminished health-related quality of life at the societal level (58). MASH, a severe form of MASLD, is a leading cause of progression to cirrhosis and HCC, becoming an increasingly common indication for liver transplantation (59). Liver cancer ranks as the second leading cause of life expectancy loss among all cancers worldwide (60), underscoring the critical need for effective progression monitoring of MASLD to HCC. However, the surveillance of MASLD-HCC progression presents challenges. Firstly, the incidence of MASLD-HCC is lower than that of viral HCC, which impacts the cost-effectiveness of screening programs (14). Secondly, MASLD is typically asymptomatic in its early stages, with clinical symptoms becoming apparent only as the condition progresses to more severe stages, such as cirrhosis or HCC. Thirdly, the presence of subcutaneous fat and hepatic steatosis in obese patients can impede the effectiveness of ultrasound imaging (61). Loomba et al. (62), after reviewing the available literature, recommended monitoring patients with advanced liver fibrosis and cirrhosis associated with MASLD but advised against monitoring MASLD patients without signs of advanced liver fibrosis. In scenarios where ultrasound conditions are challenging, CT or MRI scans are recommended as alternative imaging modalities. Specifically, non-enhanced MRI is favored over ultrasound for its brief time requirement, absence of contrast agent need, and superior sensitivity and specificity (63). The current Japanese guidelines for MASLD/MASH suggest a two-step risk stratification process, which includes screening for fibrosis through serum markers or platelet count, the Fibrosis-4 index, or the MASLD fibrosis score (64). Depending on the risk of fibrosis, subsequent liver elastography or biopsy is suggested. The 2024 Chinese Standard for the Diagnosis and Treatment of Primary Liver Cancer recommends the combination of ultrasound and serum AFP levels to screen high-risk groups early. This approach is especially significant in diagnosing patients with metabolic dysfunction-associated cirrhosis, facilitating the early detection of high-risk HCC cases (65). Despite these recommendations, appropriate monitoring strategies are still lacking, leading to MASLD-HCC often being diagnosed at a late stage. This situation highlights the urgent need for improved diagnostic and monitoring approaches to address this growing public health concern effectively. The references for diagnosing and monitoring fibrosis in MASLD were presented in Table 2. The references for diagnosis and monitoring of HCC at any stage (Table 3).

**Table 2:** Diagnosis and monitoring of fibrosis in MASLD.

**Instrument/indicator**	**Sensitivity**	**Specificity**	**Monitoring results**	**References**
FIB-4	Threshold = 2.67, sensitivity = 26.6% Threshold = 3.25, sensitivity = 96.5	Threshold = 2.67, specificity = 31.8% Threshold = 3.25, specificity = 96.0%	Higher fibrosis stages show improved diagnostic accuracy	(66–69)
NFS	Threshold = 2.0, sensitivity = 76% Threshold = 1.445, sensitivity = 61%	Threshold = 2.0, specificity = 72% Threshold = 1.445, specificity = 70%	As above	(66–68, 70)
APRI	Threshold = 1.0, sensitivity = 50% Threshold = 1.5, sensitivity = 84%	Threshold = 1.0, specificity = 18.3% Threshold = 1.5, specificity = 96.1%	As above	(66–68, 71)
MRI	79.1%	27.9%	MRI shows significantly higher accuracy compared to ultrasound	(63, 72, 73)
TVS	77.1%	25.0%	As above	(63, 72)
ELF	Threshold = 9.8, sensitivity = 70% Threshold = 10.5, sensitivity = 67%	Threshold = 9.8, specificity = 64% Threshold = 10.5, specificity = 78%	Clinically significant fibrosis (stage 2 or higher), advanced fibrosis (stage 3 or higher), and cirrhosis (stage 4) each had values of 0.8 or greater.	(74, 75)
Pro-C3	Threshold = 20.1, sensitivity = 46.7% Threshold = 25, sensitivity = 42.5%	Threshold = 12.8, specificity = 36.3% Threshold = 13.6, specificity = 34.6%	As above	(75, 76)
NIS4	Threshold = 0.8, sensitivity = 46.2% Threshold = 0.9, sensitivity = 37%	Threshold = 0.3, specificity = 57.8% Threshold = 0.5, specificity = 46%	NIS4 has met the established criteria for further qualification efforts, applicable to diagnostic enrichment for MASH, high MAS, and at-risk MASH.	(75)
FAST	Threshold = 0.35, sensitivity = 89% Threshold = 0.67, sensitivity = 49%	Threshold = 0.35, specificity = 64% Threshold = 0.67, specificity = 92%	FAST is used for non-invasive identification in patients at risk of progressive MASH during clinical trials or treatment.	(77)
MAST	Threshold = 0.165, sensitivity = 90% Threshold = 0.242, sensitivity = 75%	Threshold = 0.165, specificity = 72.2% Threshold = 0.242, specificity = 90%	The MAST score is an accurate MRI-based serum score that excels in non-invasively identifying patients at high risk of fibrotic MASH, surpassing previous scores.	(78)

FIB-4, FIBROSIS-4 Index; NFS, MAFLD fibrosis score; APRI, AST to Platelet Ratio Index; MRI, magnetic resonance imaging; TVS, transient elastography; ELF, enhanced liver fibrosis; Pro-C3, procollagen III N-terminal peptide; FAST, FibroScan-AST score; MAST, MRI-based.

**Table 3:** Diagnosis and monitoring of HCC at any stage.

**Instrument/indicator**	**Sensitivity**	**Specificity**	**Monitoring results**	**References**
AFP	Threshold = 10, sensitivity = 68.8%	Threshold = 10, specificity = 88.1%	AFP has emerged as the most commonly used marker for diagnosing HCC	(79)
AFP-L3	Threshold = 10, sensitivity = 64.2%	Threshold = 10, specificity = 91.5%	Even in the early stages of HCC, particularly when the tumor is supplied by the hepatic artery, malignant liver cells produce AFP-L3, a highly specific marker for HCC	(79, 80)
PIVKA-II	Threshold = 7.5, sensitivity = 57.2%	Threshold = 7.5, specificity = 95%	It is an abnormal prothrombin resulting from disrupted vitamin K metabolism in liver cells	(79)
GPC-3	Threshold = 272.5, sensitivity = 75	Threshold = 272.5, specificity = 81.8	The expression of GPC-3 continued to increase as HCC progressed	(81)
AFU	Threshold = 25, sensitivity = 87.5	Threshold = 25, specificity = 98.0	The dynamic curve of serum AFU activity is extremely significant for assessing the treatment effects of liver cancer, estimating prognosis, and predicting recurrence	(82)
GP73	Threshold = 78.1, sensitivity = 73.4	Threshold = 78.1, specificity = 80.0	The expression level of GP73 in primary HCC was found to be positively correlated with the degree of tumor differentiation	(83)
MRI	79.1%	27.9%	MRI shows significantly higher accuracy compared to ultrasound	(63, 72, 73)

AFP, alpha fetoprotein; AFP-L3, Lens culinaris-agglutinin-reactive fraction of AFP; AFU, Alpha-L-fucosidase; GP73, Golgi glycoprotein 73; GPC-3, glypican-3; PIVKA-II, protein induced by vitamin K absence or antagonist-II, PIVKA-II is also known as DCP.

## 5 Treatment of MASLD-HCC

**Figure 2:**
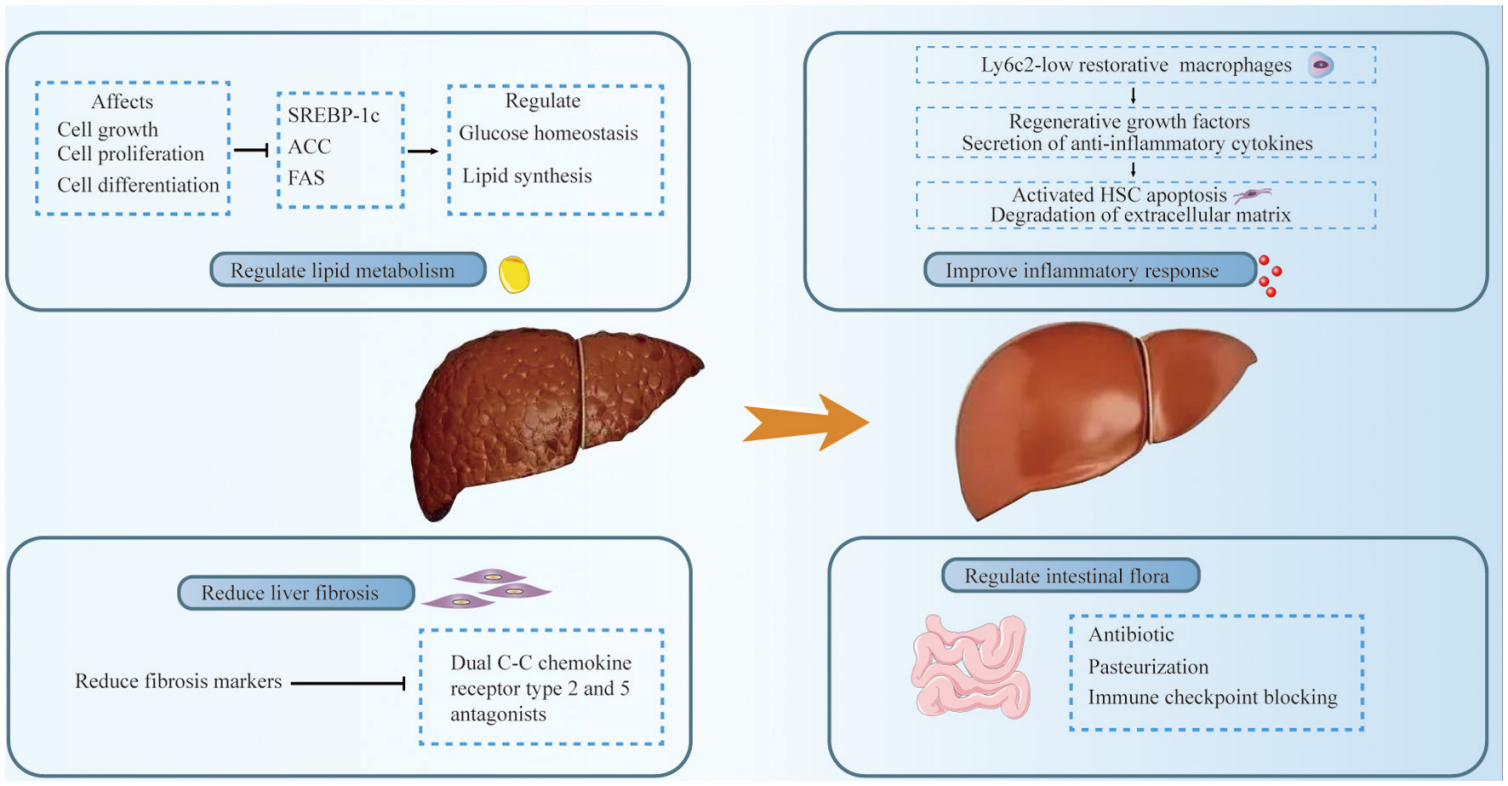
Treatment of MASLD-related HCC includes the following aspects: regulation of lipid metabolism, improvement of inflammatory response, mitigation of liver fibrosis, and intestinal microbia-related therapy. Glucose homeostasis and lipid synthesis were regulated by down-regulating SREBP-1C and ACC. Ly6c2 low-expressing restorative macrophages express regenerative growth factors and secrete anti-inflammatory cytokines. These cytokines may promote the apoptosis of activated hepatic stellate cells (HSCs) and the degradation of the extracellular matrix, thereby facilitating the resolution of inflammation. A dual C-C chemokine receptor type 2 and 5 antagonists significantly improved liver fibrosis, and antibiotics and pasteurization are used to regulate gut microbiota.

The treatment options for HCC can be broadly categorized into surgical resection and non-surgical treatments. Liver resection is the standard treatment modality for HCC patients without cirrhosis. For cirrhotic patients with HCC who meet the Milan criteria and are not candidates for primary surgical resection, liver transplantation (LT) is recommended. However, the availability of LT is constrained by the scarcity of donor organs. Current international guidelines advocate for a Barcelona Clinic Liver Cancer (BCLC)-based treatment algorithm for managing HCC (84). Despite these established guidelines, the specific impact of MASLD as an etiology on the outcomes of HCC patients remains relatively underexplored. However, it is essential to consider the unique pathophysiological mechanisms underlying MASLD-related HCC when determining treatment strategies. This approach allows for tailoring interventions according to the mechanism of disease occurrence, potentially improving patient outcomes by addressing the distinct characteristics of HCC that arises in the context of MASLD. Incorporating the understanding of MASLD's role in HCC development into treatment planning is crucial for optimizing therapeutic efficacy and patient care (Figure 2).

### 5.1 Regulation of lipid metabolism

Prevention and control of liver diseases, including HCC, can be effectively managed by regulating lipid metabolism. Changes in lipid metabolism play a significant role in the progression of many tumor cells by influencing critical cellular processes such as growth, proliferation, differentiation, and migration. Agonists that target the thyroid hormone receptor (THR)-β, predominantly located in the liver, have been shown to enhance lipophagy, mitochondrial biogenesis, and mitophagy. This stimulation leads to increased β-oxidation of fatty acids in the liver, which reduces the accumulation of lipotoxic lipids (85). Additionally, these agonists facilitate the uptake of low-density lipoprotein (LDL) and positively influence lipid profiles. Several natural compounds have been identified as potent regulators of lipid metabolism, particularly through their impact on the expression of genes related to adipogenesis (86). For instance, compounds such as lignocaine (87), oleanolic acid (88), and oxymatrine (89) have been shown to regulate glucose homeostasis and lipid synthesis by decreasing the expression of SREBP-1c, ACC, and FAS. Furthermore, betaine has demonstrated significant improvements in hepatic steatosis in C57BL/6J mice by activating AMPK and downregulating SREBP-1c. Moreover, betaine reduces hepatic triglyceride accumulation by lowering methylation of the PPARα promoter and enhancing PPARα expression, offering a promising approach to mitigating liver disease progression through the modulation of lipid metabolism (90). In the context of MASLD, research indicates that metformin modulates the ATP/AMP ratio to activate AMPK, which in turn regulates lipid metabolism (91). In HepG2 cells exposed to palmitate, metformin orchestrates the regulation of caspase-3, eukaryotic initiation factor-2a (eIF2a), and insulin receptor substrate-1 (IRS-1), thereby mitigating endoplasmic reticulum (ER) stress (92). These findings highlight the therapeutic potential of targeting lipid metabolism pathways in the prevention and management of liver diseases, including HCC, through natural compounds.

### 5.2 Modulating the inflammatory response

Inflammation plays a critical role in MASH and is considered a key factor in the progression from MASH to HCC. Consequently, anti-inflammatory strategies may serve as vital components in the treatment of both MASH and MASH-related HCC. Recent data suggest that targeting anti-inflammatory and regressive pathways shows promise for treating MASH. Evidence from patients with chronic liver diseases and murine model studies have conclusively demonstrated that fibrosis, even at advanced stages, can regress following the elimination of the underlying cause (93, 94). The activation of Mucosal-Associated Invariant T (MAIT) cells and the subsequent phenotypic shift of liver macrophages are marked pathological features of liver fibrosis, which can be targeted through anti-fibrogenic therapy (95). Specifically, the role of Kupffer cells (K.C.s) and infiltrating macrophages in mediating local liver inflammation repair is noteworthy (96). Within the liver microenvironment, K.C.s can be categorized into two primary phenotypes: pro-inflammatory M1 macrophages and anti-inflammatory M2 macrophages. Upon cessation of liver injury, hepatic macrophages transition into Ly6c2-low restorative macrophages, characterized by the expression of regenerative growth factors and the secretion of anti-inflammatory cytokines. These cytokines may promote the apoptosis and extracellular matrix degradation of activated hepatic stellate cells (HSCs), thus facilitating the resolution of inflammation (97). FGF21 inhibits hepatic lipid influx and accumulation through combined endocrine and autocrine signaling pathways, thereby preventing Kupffer cell activation and reducing the presence of lipid-associated and scar-associated macrophages to suppress fibrogenesis (98). The timely and appropriate modulation of macrophage phenotype is essential for successfully resolving inflammation and fostering liver tissue repair. Moreover, there is a notable link between anti-inflammatory approaches and antioxidants, suggesting that antioxidant treatment can also be beneficial. For example, rhodopsin has been shown to inhibit LPS-induced expression of pro-inflammatory cytokines in macrophages, reducing inflammatory cell infiltration and ameliorating liver function. Additionally, flavonoids have been found to significantly mitigate inflammation and thereby prevent hyperlipidemia and liver injury. This is achieved by enhancing antioxidant enzyme activity and inhibiting the secretion of inflammatory cytokines (TNF-α, IL-1, IL-6) in high fructose-induced obesity mice (99). These findings underscore the potential of integrating anti-inflammatory and antioxidant therapies in managing MASH and preventing its progression to HCC.

### 5.3 Reducing the progression of liver fibrosis

Fibrosis is a critical and often inevitable process in the progression of MASLD to HCC, with longitudinal studies highlighting an elevated risk of HCC and mortality in MASLD/MASH patients with severe liver fibrosis compared to those with milder forms of liver fibrosis (100). Although the progression from MASH to HCC does not invariably require liver fibrosis, the onset of excessive fibrosis almost certainly leads to irreversible liver damage. Thus, preventing and controlling the development of liver fibrosis is of paramount importance. Cysteine aspartate protease has been identified as a protease linked to hepatic apoptosis and inflammation. However, treatments with pan-cysteine protease inhibitors have not shown efficacy in improving fibrosis or in the regression of MASH (101). In the 2-year phase IIb CENTAUR trial, a dual C-C chemokine receptor type 2 and 5 antagonists demonstrated a fibrosis improvement of ≥1 stage without worsening. Interestingly, a significant proportion (60%) of patients who showed a fibrosis response after 1 year of treatment compared with placebo maintained this fibrosis reduction into the second year. Despite these promising results, the long-term effects of this treatment on fibrosis remain to be fully elucidated (102). Treatment may exert a protective effect against fibrosis by reducing the expression of fibrosis markers in mice with steatosis induced by a combination of a high-fat diet (HFD) and carbon tetrachloride (CCl4). This emerging evidence underscores the potential of novel therapeutic agents in mitigating fibrosis and highlights the necessity for continued research into effective treatments for liver fibrosis in the context of MASLD and MASH.

### 5.4 Gut microbiota interventions

The critical role of gut microbiota in the pathogenesis of MASH and its progression to hepatocellular carcinoma (MASH-HCC) has been increasingly recognized, prompting investigations into various therapeutic approaches. These include the use of antibiotics such as rivastigmine, probiotics, farnesoid X receptor (FXR) agonists, Toll-like receptor (TLR) antagonists, and inhibitors of bacterial metabolites (103). Furthermore, recent studies have explored the gut microbiome's potential role in modulating responses to immune checkpoint inhibitor therapy, highlighting a novel area of therapeutic intervention. An intriguing outcome emerged from a clinical trial involving overweight or obese insulin-resistant participants. The daily administration of pasteurized bacteria over 3 months was found to be safe, well-tolerated, and resulted in improved insulin sensitivity and lipid profiles among the treated patients. While this study did not directly demonstrate a clinical benefit in reducing HCC risk, the approach suggests a possible efficacy in lowering HCC risk among patients with MASLD (104). This finding introduces a promising avenue for leveraging gut microbiota modulation to enhance metabolic health and potentially reduce cancer risk. Additionally, a pioneering clinical trial that combines vancomycin therapy with immune checkpoint blockade has been initiated at the National Cancer Institute (NCT03785210). This trial aims to investigate whether the integration of checkpoint inhibition with selective manipulation of the microbiota yields benefits for patients with HCC (105). Immune checkpoint inhibitors, which target immunomodulatory molecules (or their ligands) on T cell surfaces to amplify antitumor immune responses, have gained approval for treating HCC (106). This innovative approach underscores the intersection of microbiome science and immunotherapy in crafting novel treatment strategies for HCC, marking a significant step forward in our understanding and management of this complex disease.

### 5.5 Metabolic pathway interventions

There is an acknowledged link between metabolic disorders and HCC. This recognition has spurred interest in therapeutic approaches that target metabolic pathways as potential strategies for HCC treatment. It has been proposed that type 2 diabetes and hypertension, hyperlipidemia, obesity, indicated by body mass index (BMI), are key contributors to severe liver disease (107). Consequently, approaches focusing on insulin resistance and diabetes may be effective in mitigating metabolic imbalances in HCC. Contemporary antidiabetic medications that also promote weight loss could help alleviate HCC symptoms. Notable among these medications are the agonists of glucagon-like peptide-1 (GLP-1), which enhance insulin secretion in response to glucose levels (108). Metformin, an oral antidiabetic drug, is the preferred treatment for type 2 diabetes due to its high efficacy and low cost (109). It reduces the risk of HCC in patients with diabetes-related chronic liver disease by inhibiting the progression of HCC. Dipeptidyl peptidase-4 inhibitors (DPP-4 inhibitors), another class of oral antidiabetic drugs based on incretin hormones, have been shown to decrease the risk of HCC in patients with type 2 diabetes and chronic HCV infection (110). Other therapeutic targets of interest are FGF19 and FGF21, which have garnered attention for their hepatoprotective properties and beneficial effects on inflammation and fibrosis, making them promising candidates for drug development. The FGF21 analog, pegbelfermin, has demonstrated favorable metabolic effects and reduced liver fat content in MASH patients after 16 weeks of treatment (111). Similarly, the FGF19 analog, NGM282, appears to be highly effective in reducing hepatic steatosis (112). In HCC, mutations within the C-terminal domains of specific receptor tyrosine kinases lead to their persistent activation. Inhibiting this phosphorylation has dual benefits: it suppresses tumor growth and inhibits angiogenesis in HCC. Tyrosine kinase inhibitors (TKIs), which serve as multi-kinase inhibitors, are primarily used in treatment to target key receptors such as VEGF, PDGF, RAF, FGF, KIT, and RET (113). A vital pathophysiological approach includes using TKIs to curb both angiogenesis in the tumor's microenvironment and the proliferation of cells (113, 114). The main receptors affected in angiogenesis signaling by these inhibitors include VEGF, PDGF, and FGF receptors (115). Peroxisome proliferator-activated receptors (PPARs) are crucial regulators of metabolism and inflammation. In various independent experimental mouse models, the pan-PPAR agonist lanifibranor has shown improvements across multiple facets of MASLD (116). In a phase 2b trial involving MASH patients, a 1,200 mg dose of lanifibranor significantly outperformed a placebo, as evidenced by at least a two-point reduction in the SAF-A score and a notably higher percentage of patients who did not experience worsening of fibrosis (117). Exploring metabolic pathways in the management of HCC has highlighted the substantial potential for therapeutic interventions. Focusing on antidiabetic drugs and agents targeting specific metabolic receptors presents a promising approach to reducing both the incidence and severity of HCC (Table 4). Further clinical trials and research are required to fully understand the effectiveness and safety of these treatment methods across various patient populations.

**Table 4:** Potential of metabolic pathway interventions in HCC related drug research.

**Therapeutic approach**	**Drug class/type**	**Mechanism**	**References**
Antidiabetic medications	GLP-1 agonists	Enhance insulin secretion in response to glucose	(108)
	Metformin	Inhibits progression of HCC in diabetes-related liver disease	(109)
	DPP-4 inhibitors	Based on incretin hormones, reduce HCC risk in diabetic patients with chronic HCV infection	(110)
FGF analogs	FGF21 analog (Pegbelfermin)	Favorable metabolic effects, reduces liver fat	(111)
	FGF19 analog (NGM282)	Reduces hepatic steatosis	(112)
Tyrosine kinase inhibitors	TKIs (multiple kinds)	Inhibit key receptors like VEGF, PDGF, RAF, FGF, KIT, and RET	(113–115)
PPAR agonists	Lanifibranor	Pan-PPAR agonist, improves multiple facets of MASLD	(116, 117)

GLP-1 agonists, glucagon-like peptide-1 receptor agonist; DPP-4 inhibitors, dipeptidyl peptidase 4 inhibitors; FGF, fibroblast growth factor.

## 6 Preventing the occurrence of MASLD-HCC

**Figure 3:**
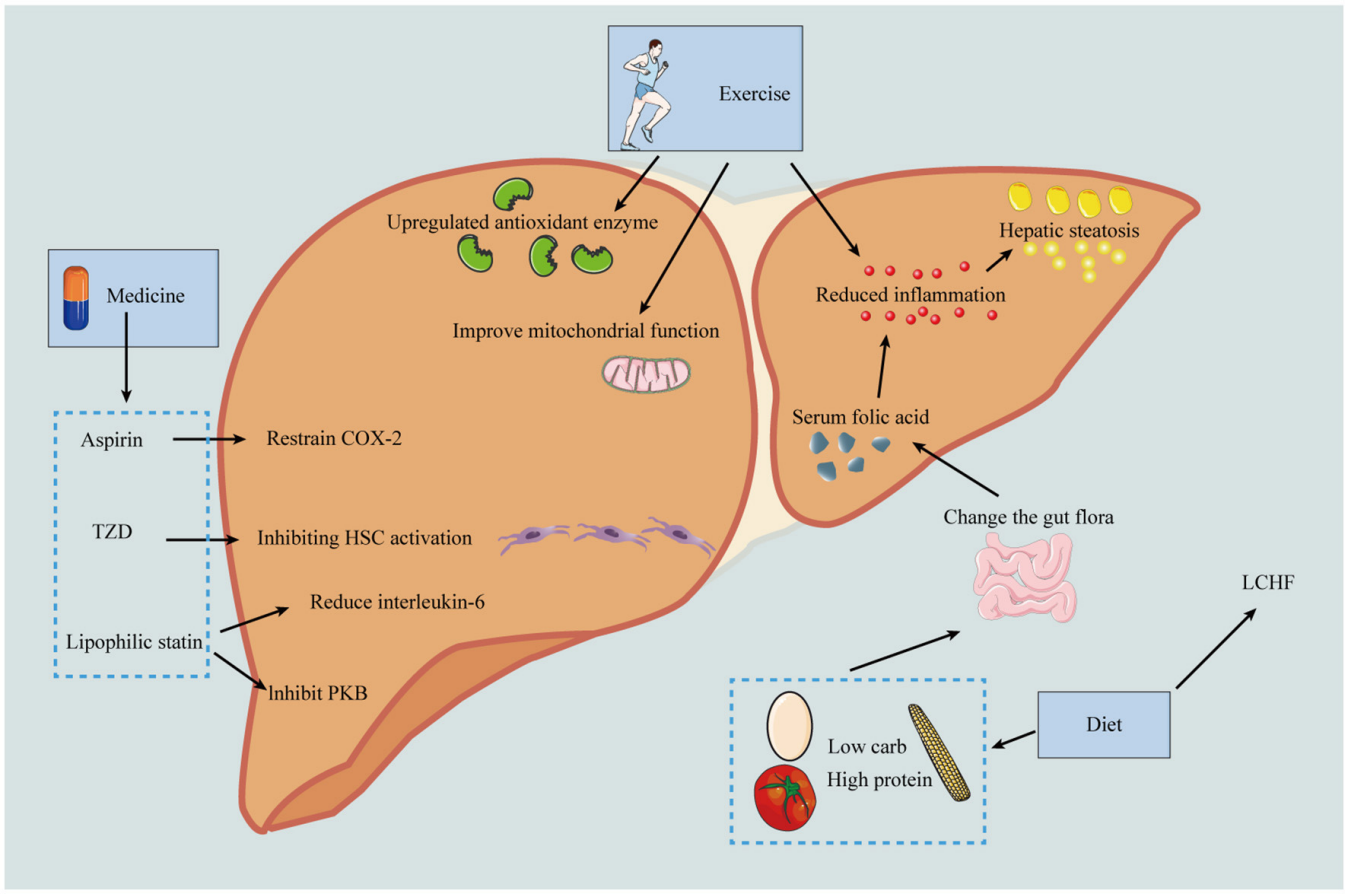
Prevention of the progression of MASLD-HCC should be carried out from the following three aspects: including diet, exercise and drugs. In terms of diet, low calorie diet can reduce the production of fat; Low carbohydrate and high protein diets reduce inflammation by improving microbiota and thereby serum folate levels, and the LCHF diet can reverse MASLD. Exercise can up-regulate antioxidant enzymes and anti-inflammatory mediators, improve mitochondrial protection, and thus reduce the risk of HCC. In terms of drugs, aspirin can selectively inhibit COX-2 and thus reduce liver fibrosis; pioglitazone can inhibit HSC activation and thus reduce HCC incidence; lipophilic statins can inhibit PKB pathway and reduce IL-6 production. HDL, high-density lipoprotein; GI, gastrointestinal; COX-2, cyclooxygenase-2; PPAR-γ, peroxisome proliferator-activated receptor gamma; MYC, MYC oncogene family; Akt, protein kinase B; NF-κB, nuclear factor kappa B; LCHF, low-carbohydrate high-fat diet.

Implementing healthy lifestyle changes, encompassing both diet and physical activity, is recognized as the most effective and cost-effective strategy for managing MASLD. This approach is pivotal not only in correcting MASLD but also in delaying its progression to HCC. Moreover, these lifestyle modifications play a crucial role in addressing other complications associated with metabolic syndrome, such as hypertension, insulin resistance, and cardiovascular risk. Additionally, adopting a healthier lifestyle can mitigate the risk of obesity-related cancers. This holistic approach underscores the significance of lifestyle interventions in the comprehensive management of MASLD and its associated comorbidities, highlighting the interconnectedness of liver health with overall metabolic and cardiovascular wellbeing. Through the promotion of dietary improvements and increased physical activity, individuals can significantly enhance their health outcomes, thereby reducing the burden of MASLD and preventing its progression to more severe liver diseases and other metabolic conditions (Table 5; Figure 3).

**Table 5:** Prevention of the progression of MASLD-HCC.

**Category**	**Intervention**	**Effect**	**References**
Daily dietary routine	Low-calorie diet	Reverses lipid autophagy	(118)
		Attenuates hepatic steatosis	
	Low-carb, high-protein diet	Improves hepatic steatosis	(118)
		Modifies gut microbiota	
		Elevates serum folate levels	
		Reduces inflammatory markers	
	LCHF	Reverses MASLD	(119–121)
		Improves prognosis by modifying cardiovascular risk factors such as total serum cholesterol and HDL levels	
Physical activity	Exercise	Enhances expression of antioxidant enzymes and anti-inflammatory mediators	(122–125)
	Vigorous exercise	Improves mitochondrial function	
		Reduces MASLD/MASH activity	
		Modulates oncogenic signaling pathways	
		Reduces risk of HCC	
Medications	Aspirin	Reduces risk of HCC without increasing GI bleeding	(126–128)
		Inhibits MASH and fibrosis through selective COX-2 inhibition	
	Pioglitazone	Decreases HCC incidence	(129–131)
		Activates PPAR-γ	
		Iinhibits hepatic stellate cell activation	
		Iinfluences lipocalin levels	
	Lipophilic statins	Inhibits MYC, Akt, and NF-κB pathways	(132, 133)
		Reduces pro-inflammatory and pro-fibrogenic cytokines production	
		Impairs tumor cell growth and invasion	

HDL, high-density lipoprotein; GI, gastrointestinal; COX-2, cyclooxygenase-2; PPAR-γ, peroxisome proliferator-activated receptor gamma; MYC, MYC oncogene family; Akt, protein kinase B; NF-κB, nuclear factor kappa B; LCHF, low-carbohydrate high-fat diet.

### 6.1 Daily dietary routine

The cornerstone of dietary intervention for managing MASLD focuses on controlling dietary caloric intake and modifying the dietary structure. Consumption of a high-fat diet has been linked to significant activation of Akt and increased mTOR phosphorylation, leading to a marked reduction in lipid autophagy. This adverse effect, however, can be mitigated through the adoption of a low-calorie diet (118). Implementing a low-carbohydrate, high-protein diet may further enhance hepatic steatosis management by modifying the gut microbiota, elevating serum folate levels, and reducing inflammatory markers. Such dietary adjustments underscore the importance of a holistic approach to MASLD management, targeting both hepatic and metabolic pathways. Moreover, cardiovascular events represent the primary cause of mortality among individuals with MASLD. A low-carbohydrate, high-fat (LCHF) diet may not only facilitate the reversal of MASLD (121) but also improve the prognosis of MASLD patients by modifying risk factors associated with cardiovascular events. These modifications include improvements in total serum cholesterol and high-density lipoprotein levels (119, 120). This dual benefit highlights the critical role of dietary management in addressing the multifaceted challenges posed by MASLD, emphasizing the potential for dietary interventions to concurrently tackle liver health and cardiovascular risk. Through strategic dietary changes, individuals can significantly impact their health outcomes, underlining the importance of diet in the comprehensive management of MASLD and its associated comorbidities.

### 6.2 Physical activity

Oxidative stress, triggered by reactive oxygen species, alongside inflammation, plays a crucial role in hepatocellular damage among patients with MASLD. Exercise emerges as a potent intervention for MASLD by enhancing the expression of antioxidant enzymes and anti-inflammatory mediators. Notably, exercise has been shown to exert a beneficial effect on MASLD (125), underlining the intrinsic value of physical activity in liver health management. Furthermore, exercise contributes to reducing the risk of HCC. This protective effect is thought to be mediated through several mechanisms, including the improvement of mitochondrial function (exemplified by enhanced mitochondrial biosynthesis and autophagy) reduction of MASLD/MASH activity, and modulation of oncogenic signaling pathways (122). The link between regular physical activity and a decreased risk of HCC was substantiated by findings from the European Prospective Investigation into Cancer and Nutrition (EPIC) study, which revealed that individuals engaging in at least 2 h of vigorous exercise weekly had a reduced HCC risk (123). This association was further supported by a meta-analysis of prospective studies, demonstrating a significantly lower risk of HCC in individuals with high levels of physical activity compared to those with low activity levels (124). Given these insights, it is advised for patients who experience improvements in fatty liver conditions due to exercise to commit to at least 12 weeks of moderate-intensity physical activity. Following this period, it becomes particularly insightful to assess the extent of hepatic steatosis, highlighting the importance of sustained exercise regimens in the effective management of MASLD and the prevention of its progression to more severe liver conditions like HCC. This recommendation underscores the critical role of exercise not only as a therapeutic strategy for MASLD but also as a preventive measure against its progression to HCC.

### 6.3 Medications

Pharmacological prevention emerges as a viable option when lifestyle interventions fail to yield desired outcomes, with several drugs demonstrating the ability to modulate risk factors and carcinogenic pathways associated with MASLD/MASH-induced HCC. A notable national registry study in Sweden provided evidence that regular intake of aspirin at doses below 160 mg/day for a duration of at least 5 years significantly reduced the risk of HCC without escalating the risk of gastrointestinal bleeding (126). Further, in a cohort of 361 patients with biopsy-proven MASLD, daily aspirin administration was significantly associated with a reduced odds ratio (OR) for MASH and fibrosis. This protective effect of aspirin against MASH and fibrosis is thought to be mediated through selective inhibition of cyclooxygenase-2 (127). Based on a systematic review and meta-analysis by Abdelmalak et al., the use of aspirin in patients without cirrhosis is associated with an ~30% reduction in the risk of HCC. However, in patients with cirrhosis, this protective effect did not reach statistical significance (adjusted HR 0.96, 95% CI 0.84–1.09). Furthermore, the study also noted an increased risk of bleeding associated with aspirin use (adjusted HR 1.11, 95% CI 1.02–1.22) (128). Given the inherently higher risk of bleeding in cirrhotic patients, this risk may be more pronounced. Therefore, the pharmacological prevention of HCC should be carefully case-selected, particularly considering the stage of liver disease and the individual bleeding risk, to ensure an optimal risk-benefit ratio. Pioglitazone, an activator of the peroxisome proliferator-activated receptor-gamma (PPAR-γ), renowned for its insulin-sensitizing properties, has been observed to decrease the incidence of HCC in both a hospital-based case-control study and a population-based cohort study (129). The anticancer effects of pioglitazone are postulated to arise from the inhibition of hepatic stellate cell activation, as suggested by *in vitro* studies (130). Moreover, pioglitazone has shown to positively influence lipocalin levels, which is linked to the prevention of carcinogenesis (131). Lipophilic statins, known for their higher lipid solubility and membrane permeability, enable them to exert cholesterol-dependent effects on HCC development (132). These effects potentially include the inhibition of key oncogenic pathways such as MYC, protein kinase B (Akt), and NF-κB, along with a reduction in the production of pro-inflammatory and pro-fibrogenic cytokines such as interleukin-6, tumor necrosis factor-alpha, and transforming growth factor-beta1. Furthermore, simvastatin has been documented to impair tumor cell growth and disrupt the adhesion of tumor cells to endothelial cell monolayers, consequently hindering tumor cell invasion (133). Especially for patients with decompensated cirrhosis and liver failure, considering the increased risk of myopathy side effect, current evidence suggests that statins should be used cautiously and only at low doses (max. 20 mg) to balance therapeutic benefits and potential adverse effects (134). This array of pharmacological interventions highlights the nuanced approach required in the management of MASLD/MASH and the prevention of its progression to HCC, where medication can play a critical role alongside lifestyle modifications.

## 7 Progress and prospects

The clinical management of HCC still faces major challenges, and many unanswered questions remain to be addressed by the scientific community. In particular, there are challenges and problems in the area of MASLD-HCC, where some of the pathogenesis is understood, but much remains to be learned. Although current treatments can target known mechanisms, completely halting the pathogenesis through pharmacological or surgical interventions remains elusive. Consequently, the emphasis on prevention (particularly through diet and exercise) is paramount. This review delves into the pathogenesis, diagnosis, and monitoring of MASLD-HCC, alongside treatment options and preventative measures. Presently, the underlying mechanisms of MASLD-HCC are still under investigation, with a notable deficiency in effective screening tools, preventive measures, and curative medications. Hence, it is imperative for both patients and physicians to prioritize the management and control of relevant risk factors in clinical practice. Such diligence is essential to offer precise medication guidance and support patients in achieving a more favorable prognosis. Emphasizing preventative strategies, including lifestyle modifications, can play a crucial role in mitigating the risk of MASLD progressing to HCC. This approach underscores the need for continued research and development in the field, aiming to enhance our understanding, refine diagnostic tools, and discover more effective treatments for this complex disease.

## Author contributions

YM: Conceptualization, Writing—original draft, Writing—review & editing, Visualization. JW: Writing—original draft, Data curation, Methodology, Formal analysis, Investigation, Visualization, Writing—review & editing. WX: Data curation, Writing—original draft, Formal analysis, Methodology, Visualization. XF: Conceptualization, Funding acquisition, Supervision, Validation, Writing—review & editing.
